# Antimicrobial Resistance in *Campylobacter*

**DOI:** 10.3201/eid1106.040689

**Published:** 2005-06

**Authors:** Louis Anthony Cox, Dennis Copeland, Michael Vaughn

**Affiliations:** * Cox Associates, Denver, Colorado, USA;; † Bayer HealthCare, Shawnee, Kansas, USA

**Keywords:** campylobacter, risk analysis, antimicrobial risk assessment

**To the Editor:** Iovine and Blaser (1) write, "This therapeutic use [of enrofloxacin] was withdrawn (2) but is now under appeal" and "Despite the restrictions on enrofloxacin use, emergence of fluoroquinolone-resistant *Campylobacter* species, with poultry as an important source, has been documented in the United States… Therefore, our conclusion remains: use of enrofloxacin in poultry materially contributed to increase in human infection by fluoroquinolone-resistant *Campylobacter* species."

These claims propagate the following important errors. First, the therapeutic use of enrofloxacin was not withdrawn. Judge Davidson's order to withdraw the approval was an initial decision, to which exceptions were filed in 2004. A final decision rests with the US Food and Drug Administration Commissioner.

Second, poultry has not been identified as an important source of fluoroquinolone resistance in human *Campylobacter* isolates. The raw data of the cited Smith et al. article (3) indicate a nonsignificant negative association between chicken consumption and fluoroquinolone resistance in human isolates. Substantial resistance levels in Northern Hemisphere countries with and without enrofloxacin use, which occurred well before fluoroquinolones were ever used in animals (3–5), also suggest that attribution of such resistance to enrofloxacin is simplistic.

Finally, rational decision-making is based on probable future consequences of a decision, not past history or causes of the current situation. Iovine and Blaser's claim, "Thus the decision to withdraw therapeutic use of enrofloxacin (3) was warranted," is not implied, even if enrofloxacin use caused the emergence of fluoroquinolone resistance. If withdrawing enrofloxacin increases campylobacteriosis from airsacculitis-positive chickens, withdrawal may greatly harm human health. A rational withdrawal decision cannot be justified. In summary, Iovine and Blaser's view that enrofloxacin should be banned is not supported by the data that they have cited or by principles of sound risk management and decision-making.
