# Supplementary material for: Antimicrobial Resistance in Campylobacter
Source: Emerg Infect Dis. 2005 Jun;11(6):983–4. doi: 10.3201/eid1106.040689 (PMC3367608; doi:10.3201/eid1106.040689)
Supplement: Related texts

# Reply: Antimicrobial Resistance in *Campylobacter*

**DOI:** 10.3201/eid1106.050266

**Authors:** Nicole M. Iovine, Martin J. Blaser

**Affiliations:** * New York University School of Medicine, New York, NY, USA;; † New York Harbor Department of Veterans Affairs Medical Center, New York, NY, USA; 1 New Animal Drug Application

**To the Editor:** Cox and colleagues raised 3 major points. For the first point, we stated (1) "This therapeutic use was withdrawn but is now under appeal." The actual language of US Federal Drug Administration Judge Davidson's ruling is "Enrofloxacin found not shown to be safe under the conditions of use upon the basis of which the application was approved as required under § 512(e)(1)(B) of the Federal Food, Drug, and Cosmetic Act (the Act) [21 U.S.C. § 360 b(e)(1)(B)]. Approval of NADA^1^ for enrofloxacin ordered withdrawn" (1). The drug manufacturer now is appealing the ruling.

For the second point, the authors state that poultry has not been identified as an important source of fluoroquinolone resistance in human *Campylobacter* isolates. In both Denmark and Spain, introduction of fluoroquinolones into poultry led to a rapid rise in resistance to *Campylobacter* in both poultry and human isolates (2–5), and banning their use in Denmark led to a rapid fall in resistance (6). Cox and colleagues may maintain that there is no "proof of a causal relationship," but the relationship is sufficiently strong, temporally restricted, biologically plausible, and coherent to convince disinterested observers, including Judge Davidson and ourselves, otherwise.

For the third point, that decisions must consider probable consequences, we agree. However, Cox et al. appear to use "possible" as their standard. In fact, nearly everything is possible, including the reasoning that they offer. However, in our opinion, based on experience as scientists and microbiologists, we deem the possible consequences described by Cox et al. as insubstantial compared to the clear and present danger to human health of continuing fluoroquinolone use in poultry. Obfuscation and delay have been effective tactics used to maintain profitability even when the facts indicate a different course of action. We hope that the FDA Commissioner will carefully weigh the actual evidence of the risk to human health imposed by the use of fluoroquinolones in poultry.

## References

[1] Daniel J. Davidson FALJ. In: The matter of enrofloxacin for poultry: withdrawal of approval of Bayer Corporation's new animal drug application 1 (NADA) 140-828 (Baytril). In: FDA Docket No 00N-1571; 2004.

[2] Endtz HP, Ruijs GJ, van Klingeren B, Jansen WH, van der Reyden T, Mouton RP. Quinolone resistance in *Campylobacter* isolated from man and poultry following the introduction of fluoroquinolones in veterinary medicine. J Antimicrob Chemother. 1991;27:199–208. 10.1093/jac/27.2.1992055811

[3] Reina J, Borrell N, Serra A. Emergence of resistance to erythromycin and fluoroquinolones in thermotolerant *Campylobacter* strains isolated from feces 1987–1991. Eur J Clin Microbiol Infect Dis. 1992;11:1163–6. 10.1007/BF019611371291314

[4] Sanchez R, Fernandez-Baca V, Diaz MD, Munoz P, Rodriguez-Creixems M, Bouza E. Evolution of susceptibilities of *Campylobacter* spp. to quinolones and macrolides. Antimicrob Agents Chemother. 1994;38:1879–82.781099310.1128/aac.38.9.1879PMC284656

[5] Velazquez JB, Jimenez A, Chomon B, Villa TG. Incidence and transmission of antibiotic resistance in *Campylobacter jejuni* and *Campylobacter coli*. J Antimicrob Chemother. 1995;35:173–8. 10.1093/jac/35.1.1737768766

[6] Use of antimicrobial agents and occurrence of antimicrobial resistance in bacteria from food animals, foods and humans in Denmark. Copenhagen: Danish Zoonosis Center, Danish Veterinary Institute; 2003.
